# Combined use of transversus abdominis plane block and laryngeal mask airway during implementing ERAS programs for patients with primary liver cancer: a randomized controlled trial

**DOI:** 10.1038/s41598-020-71477-x

**Published:** 2020-09-10

**Authors:** Hai-ming Huang, Rui-xia Chen, Lin-mei Zhu, Wen-shuai Zhao, Xi-jiu Ye, Jian-wei Luo, Fu-ding Lu, Lei Zhang, Xue-ying Yang, Yuan Yuan, Jun Cao

**Affiliations:** 1 Department of Anesthesiology, Sun Yat-Sen Memorial Hospital, Sun Yat-Sen University, Guangzhou, 510120 Guangdong China; 2 Department of Obstetrics and Gynecology, Guangdong Clifford Hospital, Guangzhou, 511495 Guangdong China; 3 Department of Hepatobiliopancreatic Surgery, Sun Yat-Sen Memorial Hospital, Sun Yat-Sen University, NO. 107, Yanjiangxi Road, Guangzhou, 510120 Guangdong China

**Keywords:** Cancer of unknown primary, Cancer therapy

## Abstract

The incidence and mortality of primary liver cancer are very high and resection of tumor is the most crucial treatment for it. We aimed to assess the efficacy and safety of combined use of transversus abdominis plane (TAP) block and laryngeal mask airway (LMA) during implementing Enhanced Recovery After Surgery (ERAS) programs for patients with primary liver cancer. This was a prospective, evaluator-blinded, randomized, controlled parallel-arm trial. A total of 96 patients were enrolled (48 in each group). Patients in the control group received general anesthesia with endotracheal intubation, while patients in the TAP + LMA group received general anesthesia with LMA and an ultrasound-guided subcostal TAP block. The primary end-point was postoperative time of readiness for discharge. The secondary end-points were postoperative pain intensity, time to first flatus, quality of recovery (QoR), complications and overall medical cost. Postoperative time of readiness for discharge in the TAP + LMA group [7 (5–11) days] was shorter than that of the control group [8 (5–13) days, *P* = 0.004]. The postoperative apioid requirement and time to first flatus was lower in the TAP + LMA group [(102.8 ± 12.4) µg, (32.7 ± 5.8) h, respectively] than the control group [(135.7 ± 20.1) µg, *P* = 0.000; (47.2 ± 7.6) h, *P* = 0.000; respectively]. The QoR scores were significantly higher in the TAP + LMA group than the control group. The total cost for treatment in the TAP + LMA group [(66,608.4 ± 6,268.4) CNY] was lower than that of the control group [(84,434.0 ± 9,436.2) CNY, *P* = 0.000]. There was no difference in complications between these two groups. The combined usage of a TAP block and LMA is a simple, safe anesthesia method during implementing ERAS programs for patients with primary liver cancer. It can alleviate surgical stress, accelerate recovery and reduce medical cost.

## Introduction

Since first reported by professor Kehlet in 1997, the ERAS program has been globally acknowledged and widely used in clinic^1–3^. By a series of perioperative optimal interventions based on evidence, the implementation of ERAS can effectively reduce surgical stress, accelerate postoperative recovery and decrease morbidity as well as medical cost^4,5^. Anesthesiologists play a key role in implementing ERAS, especially in regulating surgical stress and postoperative pain. However, the specific measures of ERAS for patients with primary liver cancer are still uncertain in China. Regional blockade combined with general anesthesia is recommended strongly as the optimal anesthetic protocol for open abdominal surgery with an ERAS program^6,7^.

The methods of regional blockade include epidural block, peripheral nerve block, and local wound infiltration. The analgesic effect of an epidural block is perfect, but common contraindications and underlying catastrophic complications limit its application in liver surgery^7,8^. Local wound infiltration is also not the preferred regional blockade for liver surgery, because of short analgesic time, susceptibility to systemic poisoning from local anesthetic and adverse effect on wound healing. Peripheral nerve block techniques that can be used for open liver surgery include thoracic paravertebral block (TPVB), intercostal nerve block, quadratus lumborum block (QLB), transversus abdominis plane (TAP) block and rectus sheath block. TPVB, intercostal nerve block and QLB are difficult to popularize during ERAS because of high difficulty and risk of their procedures. The rectus sheath block only has a blocking effect on the nerves innervating the antetior abdominal wall, so it is not suitable for open liver surgery when used alone. TAP block is a regional blockade developed in the past decade which can provide satisfactory analgesia for abdominal surgery^9^. Guided by ultrasound, TAP block is quite simple and safe to perform, with almost no contraindications. Therefore, TAP block can be conveniently used in abdominal surgery with ERAS programs.

Endotracheal intubation (ETI) is the most commonly used method of airway management during general anesthesia, but it usually leads to a strong stress response or even airway injury. A laryngeal mask is a supraglottic ventilation device which has little noxious stimulation to the airway and may increase comfort of patients after general anesthesia^10^. The inventions of some newer laryngeal masks, like the double-tube laryngeal mask or flexible laryngeal mask, have solved the problem of misplacement or leakage which sometimes occur with traditional laryngeal masks^11^. Thus, laryngeal masks are becoming more popular. In Europe and America, the usage of laryngeal mask is close to or even exceeds that of endotracheal tube (ETT). Therefore, we hypothesized that the combined usage of a TAP block and LMA could be safely performed for patients with primary liver cancer during implementing ERAS programs and reduce the postoperative time of readiness for discharge.

## Methods

### Study design

This prospective, evaluator-blinded, randomized, controlled parallel-arm trial was conducted at Sun Yat-sen Memorial Hospital, Sun Yat-sen University, China. The study protocol was approved by the Medical Ethics Committee of Sun Yat-sen Memorial Hospital of Sun Yat-sen University (Approval number: 201806). All procedures were performed in accordance with the declaration of Helsinki. Written informed consent was obtained from every patient before randomization.

### Patients

Patients with primary liver cancer who were scheduled for open hepatectomy with ERAS programs (Table 1) were evaluated for eligibility. Inclusion criteria were as follows: age between 18 and 65 years; body mass index of 18.5–28 kg/m^2^; number of tumor less than 3; tumor’s maximum diameter or sum of diameters smaller than 10 cm; class A or B of Child–Pugh liver function; tumor’s clinical stage as I or II; the remaining liver volume/standard liver volume ratio of > 40%; no difficulty of intubation or inserting an LMA; American Society of Anesthesiologist physical status classification between I and III. Exclusion criteria were as follows: presence of comorbidities such as diabetes, pathological cardiopulmonary disease and renal insufficiency; a New York Heart Association class of heart function of III or greater; intrahepatic vascular invasion or extrahepatic metastasis of tumor seen by computerized tomography (CT) or magnetic resonance imaging (MRI); having received chemotherapy or radiotherapy before surgery; having undergone abdominal surgery; infection of the site of TAP block; allergy to any study medication; a history of alcohol or apioid abuse; difficulty of communication with family or hospital staff. After randomization, patients who experienced a failure of TAP block or LMA ventilation, suffered systemic poisoning from local anesthetic, were found to have extensive metastasis of tumor after laparotomy, lost blood over 1,000 ml during surgery, underwent a long operating time of over 6 h, needed to be transferred into intensive care unit (ICU) after surgery, or refused to go on participating in this trial were excluded from the analysis.

**Table 1:** The ERAS program adopted in this trial.

**Preoperative procedures**
Preoperative assessment, education and psychological counseling by talk, text and caption
Preparation before admission: cessation of smoking and alcohol for 14 days, quick walk for 30 min daily, oral enteral nutritional powder if plasma albumin ≤ 30 g/L, infuse red blood cell if Hb < 70 g/L
No preoperative use of sedatives or anticholinergic drugs
No bowel preparation, fasting for 6 h, and oral 10% glucose 500 ml 2 h before surgery
No routine placement of gastric tube; if necessary, remove it as soon as possible
Antibiotic prophylaxis 30 min before surgery
Use heparin to prevent deep vein thrombosis for high risk patients and monitor coagulation
**Intraoperative procedures**
Continuously monitor body temperature and maintain its stability by warmed blanket, warmed infusion and preheated peritoneal irrigation
Infuse crystal liquid mainly and restrict the volume (CVP ≤ 10 mmHg); rapidly infuse 200–300 ml colloidal fluid if severe hypotension occurs during the implementation of controlled-low CVP
No routine placement of peritoneal drainage tube; if necessary, remove it within 48 h
Anesthesia method: ETI general anesthesia for the control group, LMA general anesthesia combined with a subcostal TAP block for the TAP + LMA group
Use of short-acting anesthetics (i.e. remifentanil, sufentanil, propofol and sevoflurane)
Monitor anesthetic depth and maintain its stability
Intravenous tropisetron 10 mg for preventing postoperative nausea and vomiting
**Postoperative procedures**
Multimodal postoperative analgesia: a. a single-shot regional block (local wound infiltration for the control group and a subcostal TAP block for the TAP + LMA group); b. patient-controlled intravenous analgesia (PCIA) with sufentanil; c. intravenous parecoxib 40 mg twice daily for 3 days; d. oral analgesic
Assess pain intensity with numerical rating scale (NRS) and inject additional analgesic when NRS score ≥ 5
Intravenouslydrip dexamethasone 5 mg daily for 3 days to alleviate inflammation
Treatment of postoperative nausea and vomiting
Remove drainage tube and urinary tube as soon as possible
Early oral intake: a little water on the day of surgery; liquid diet on 1st postoperative day; semi-liquid diet on 2nd postoperative day; normal diet from 3rd postoperative day on
Early mobilization: exercise in bed on the day of surgery; walk in the ward at least twice on 1st postoperative day; walk at least 4 times on 2nd postoperative day; continuously walk from 3rd postoperative day on
Use low molecular weight heparin to prevent deep vein thrombosis

### Randomization and blinding

This study was an observer-blinded, randomized control trial. Randomization, stratified by surgeons, was performed by independent personnel (Zhu L), through two random digit tables generated by computer software. The random digit and the information of group allocation for every enrolled patient was sealed with an opaque envelope. Two anesthesiologists (Chen R, Yang X) performed all the intraoperative assessments, and two other anesthesiologists (Yuan Y, Lu F) performed all the postoperative assessments; two surgeons (Cao J, Zhang L) performed local infiltration and assessed if the patients had achieved the standard of hospital discharge, and two independent persons (Zhao W, Ye X) performed the statistical analysis of the data. All these investigators were blinded to the group allocation. The solutions for local infiltration were all configured by the same anesthesia nurse (Luo J), who knew the group allocation. All procedures of TAP blockades and intubation or insertion of LMAs were performed by the same anesthesiologist (Huang H), who knew the group allocation.

**Figure 1:**
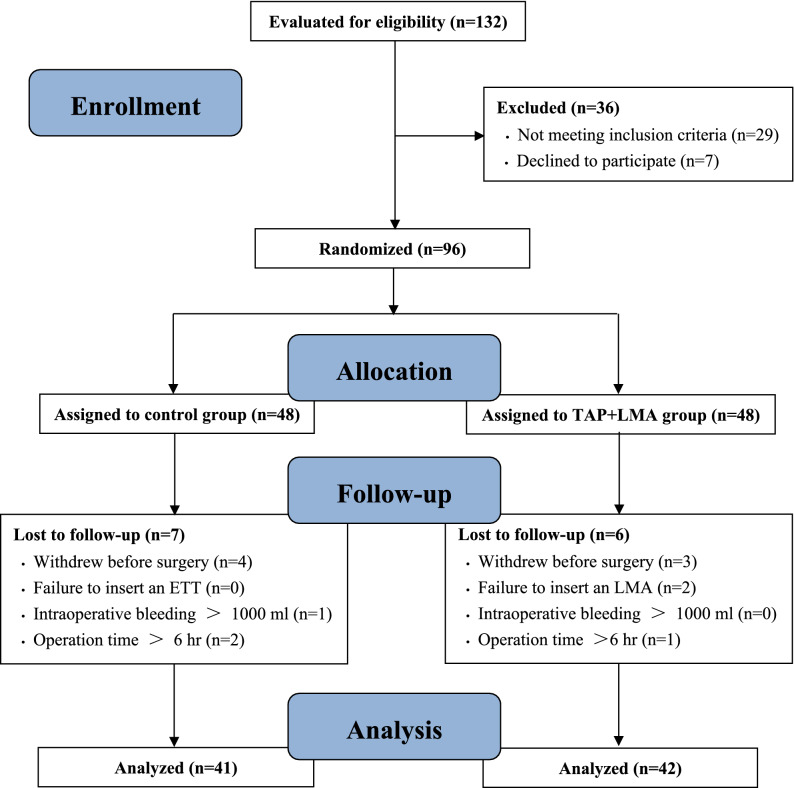
Flow diagram for this trial.

A total of 132 patients scheduled for open hepatectomy with an ERAS program were initially evaluated for eligibility between January and December 2019. 36 patients were excluded for not meeting the inclusion criteria or refusal to participate in the study. Finally, 96 eligible patients were randomly divided into two groups (n = 48), according to different interventions. During the implementation of this trial, 7 patients were excluded in the control group (4 withdrew prior to surgery, 1 had excessive bleeding of over 1,000 ml intraoperatively and 2 exceeded the operation time of more than 6 h), while 6 patients were excluded in the TAP + LMA group (3 withdrew prior to surgery, 2 failed in LMA insertion and 1 exceeded the operation time of more than 6 h). Therefore, 41 remained in the control group and 42 remained in the TAP + LMA group. This data wasthen statistically analyzed (Fig. 1).

### Interventions

**Figure 2:**
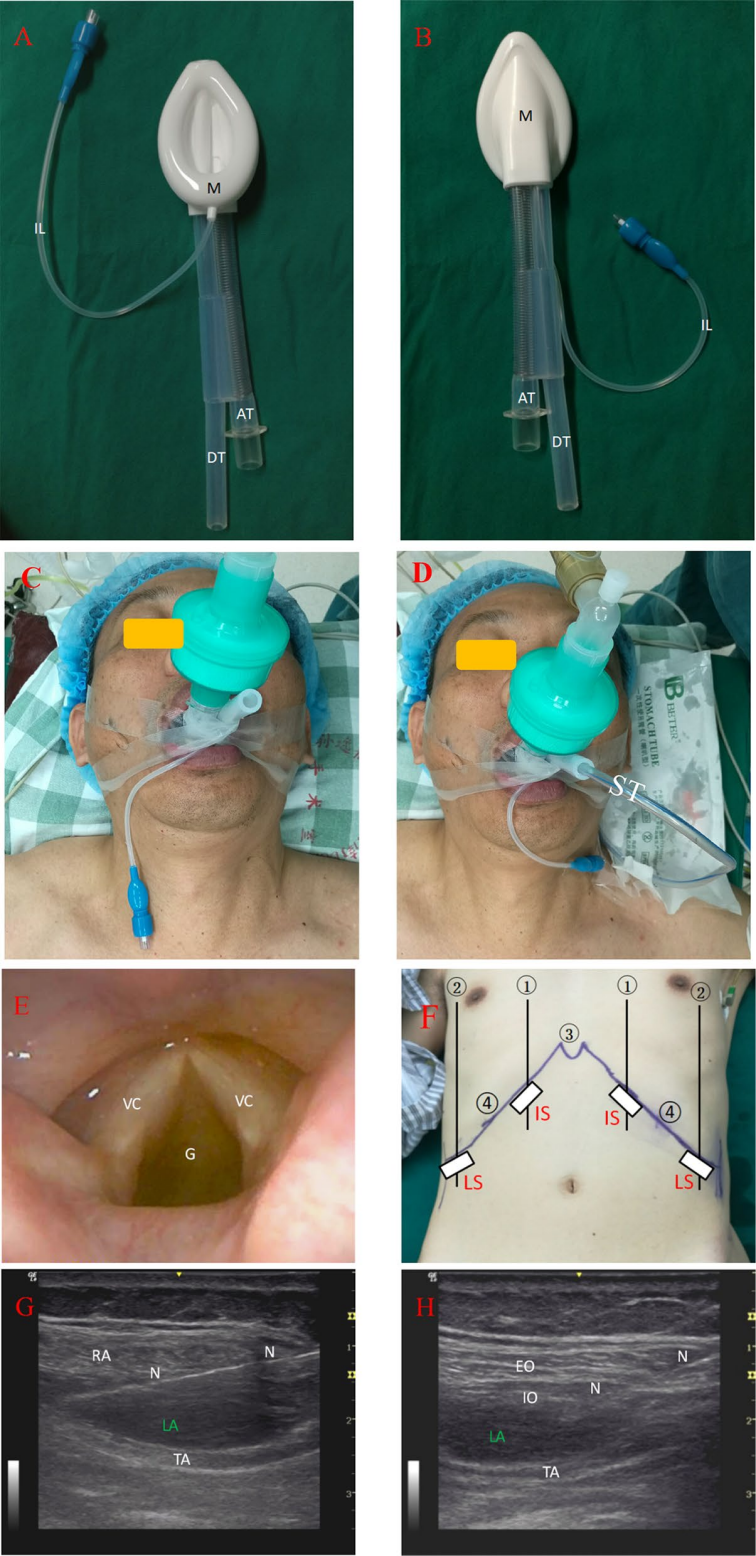
Procedures of LMA ventilation and subcostal TAP block. (**A**) Double-tube LMA: ventral side; (**B**) Double-tube LMA: back side; (**C**) Placement of LMA; (**D**) LMA ventilation and esophageal drainage; (**E**) LMA positioning by fiberoptic bronchoscopy; (**F**) positions of ultrasound probe for TAP block; (**G**) Ultrasound image of TAP block: inner site; (**H**). Ultrasound image of TAP block: lateral site. *AT* airway tube, *DT* esophageal drainage tube, *M* mask, *IL* inflation line, *ST* stomach tube, *G* glottal, *VC* vocal cord, ①: parasternal line, ②: anterior axillary line, ③: xiphoid, ④: costal arch, white rectangle: ultrasound probe, *IS* inner site, *LS* lateral site, *N* needle, *LA* local anesthetic, *RA* rectus abdominis, *TA* transversus abdominis, *EO* obliquus externus abdominis, *IO* obliquus internus abdominis.

Patients in both groups received the same ERAS program except for the different interventions. For the control group, patients were treated with endotracheal intubation for ventilation after anesthesia induction, a preoperative TAP block with 40 ml normal saline as control and local wound infiltration with 40 ml solution of ropivacaine 3 mg/kg plus dexamethasone 0.1 mg/kg at the conclusion of surgery. On the contrary, patients in the TAP + LMA group had a double-tube LMA (HZ-II, Royal Fornia Medical Equipment Co., Ltd. China-U.S. Joint Venture) inserted for ventilation after induction, had a TAP block with 40 ml solution of ropivacaine 3 mg/kg plus dexamethasone 0.1 mg/kg, and received local wound infiltration with 40 ml normal saline at the conclusion of surgery. Guided by ultrasound, a subcostal TAP block was performed bilaterally at the parasternal line and at the anterior axillary line, with 10 ml solution injected in each site (Fig. 2).

### Anesthesia and analgesia

While in the operating room, all enrolled patients were continuously monitored for standard measures including electrocardiogram, heart rate, non-invasive arterial blood pressure and oxygen saturation. Monitoring of Narcotrend Index (NI) was used for assessing patients’ depth of consciousness. Placement of a peripheral vein catheter was established for infusion. Radial artery catheterization was performed to monitor invasive arterial pressure and to measure blood gas.

After induction of total intravenous anesthesia with propofol/sufentanil/cis-atracurium, positive pressure ventilation was performed in the patients of control group by ETI and in the patients of TAP + LMA group by LMA. Auscultation of respiratory tone, observation of respiratory parameters and examination with a flexible fiberoptic bronchoscopy were used to determine whether the laryngeal mask was in correct position. If necessary, gastric tubes were placed with the help of a laryngoscope in the patients of control group or through the esophageal drainage tube of the LMA in the patients of the TAP + LMA group. Then, the procedures of ultrasound-guided subcostal TAP block were implemented as described above.

Maintenance of anesthesia was achieved by administration of sevoflurane, remifentanil and cis-atracurium. Dosages of anesthetic drugs were adjusted to maintain arterial blood pressure and heart rate fluctuating in the range of -20% to + 20% of the base value and NI fluctuating in the range of 37 to 56. Cis-atracurium was continuously infused at the rate of 0.05 mg/kg/h to maintain muscle relaxation during surgery, and an additional dose of 0.05 mg/kg would be injected intravenously if muscle relaxation was not enough (assessed by the surgeon). If necessary, cardiovascular active drugs (i.e. norepinephrine, atropine, esmolol, nicardipine) were used to maintain stable hemodynamics. Controlled-low Central Venous Pressure (CVP) technology was adopted to maintain CVP between 0 and 5 cmH_2_O during hepatectomy. This was done by restriction of fluid, control of anesthesia depth and stress response, adjustment of patients’ position, and application of vasoactive drugs (nitroglycerin and dobutamine). The CVP value was restored to normal after completing the hepatectomy. Allogeneic blood transfusion was administered if hemoglobin was less than 70 g/L. Body temperature was continuously monitored and maintained within 36–37℃ by multiple approaches including warmed infusion, application of electric blanket and preheated peritoneal irrigation.

At the conclusion of surgery, tropisetron 10 mg was administered to prevent postoperative nausea and vomiting and sufentanil 0.1ug/kg was administered to alleviate remifentanil-induced hyperalgesia. Local wound infiltration was performed with different solution in the two groups, as described above. Multimodal analgesia was used postoperatively in both groups, including regional block, patient-controlled intravenous analgesia (PCIA) with sufentanil, intravenous parecoxib 40 mg twice per day for 3 days and oral analgesic.

### Study end-points

The primary end-point of the study was postoperative time of readiness for hospital discharge. The discharge criteria included: pain score less than 4, solid diet without infusion, normal bowel movement, well-healed wound, normal organ function, normal count of white blood cell (WBC), no fever, life independence.

The secondary end-points were postoperative analgesic requirement within 48 h, time to first flatus and overall medical cost. The overall medical costs included the cost for treatment of the patient from check-in to hospital discharge. Blood glucose and lactate were tested before anesthesia (t0), at surgical exploration (t1), at the conclusion of surgery (t2) and at departure from the post-anesthesia care unit (PACU) (t3). Dosages of anesthetics and vasoactive drugs, surgery duration, anesthesia duration, hepatectomy duration, duration of hepatic blood flow occlusion, bleeding volume, infusion volume and urine volume were recorded.

The postoperative pain intensity of the surgical site was assessed with the numerical rating scale (NRS), in which 0 represented no pain and 10 represented the most severe pain. Postoperative variables such as time to removing ETT or LMA, duration of PACU stay, time to first mobilization off the bed, incidence of nausea/vomiting within 48 h and complications were also recorded and compared. The 40-item quality of recovery (QoR-40) survey was performed before surgery, at 1 and 3 days after surgery, and on the day of discharge, referred to other studies^12,13^ and described as the figure in the supplementary material (Figure S1).

### Statistical analysis

Statistical analyses were performed with SPSS software version 19.0 (IBM Corporation, Armonk, NY, USA). Continuous variables were tested if they were normally distributed by inspection of histograms. Variables with normal distribution were expressed as mean ± standard deviation and compared between groups using a 2-sample Student’s *t*-test. Data with abnormal distribution were expressed as median (range) and analyzed using non-parametric test. Blood glucose, lactate, the NRS pain score and the QoR-40 score were compared between groups using repeated-measures analysis of variance (ANOVA). Categorical data was expressed as ratio or frequency and analyzed using Chi-square test or Fisher’s exact test. Two-tailed *P* < 0.05 indicated statistical significance.

Based on our preliminary experiment consisting of 24 patients, the sample size for this trial was calculated using SAS software version 9.4 (SAS Institute Inc., Iowa City, IA, USA). The postoperative time of readiness for hospital discharge in the control group was (9 ± 2.5) days, while that of the TAP + LMA group was (7.5 ± 2.0) days. Accordingly, 42 patients were required per group to achieve 85% statistical power (1-β) at a significance level (α) of 0.05. To account for a 15% drop-out rate, we enrolled 48 patients per group finally.

### Statement

All information and images showed in this manuscript that may lead to identification of the study participants, have obtained the participants’ informed consent to publish in an online open-access publication.

## Results

The baseline characteristics of the patients were similar between the two groups (Table 2). The primary outcome (postoperative time of readiness for discharge) and secondary outcomes (postoperative apioid requirement, time to first flatus and total cost for treatment) of the TAP + LMA group were significantly lower than those of the control group (Table 3). Patients who received TAP + LMA reported significantly lower NRS pain scores at all measured time points in the 48 h following surgery. The time to first mobilization was significant shorter in the TAP + LMA group than the control group. No significant differences were observed between the two groups in the incidence of nausea or vomiting within those 48 h. There was no statistical difference of the QoR-40 scores between the two groups 1 day prior to surgery or on the day of discharge. However, QoR-40 scores of patients in the TAP + LMA group were statistically higher at 1 and 3 days after surgery.

**Table 2:** Baseline Characteristics of the Patients.

Variable	Control group (n = 41)	TAP + LMA group (n = 42)	*P*-value
Age (years)	51.4 ± 6.6	52.3 ± 6.3	0.532
Sex (male/female)	32/9	34/8	0.791
BMI (kg/m^2^)	22.3 ± 1.3	22.2 ± 1.7	0.765
ASA physical status (II/III)	29/12	28/14	0.690
Preoperative Child–Pugh classification of liver function (A/B)	31/10	34/8	0.555
Number of tumor (1/2)	28/13	33/9	0.289
Diameter of tumor (mm)	63.0 ± 17.2	64.8 ± 17.4	0.648
**Location of tumor**			
Left lateral lobe	8	10	0.635
Left inner lobe	6	7	0.799
Right anteriorlobe	18	15	0.446
Right posterior lobe	9	11	0.652
Clinical stage of tumor (I/II)^a^	20/21	25/17	0.326
**Cell type of tumor**			
HCC	32	34	0.743
ICC	5	3	0.436
HCC-ICC	4	5	0.753
**Differentiation of tumor**			
Grade I	6	8	0.591
Grade II	26	23	0.423
Grade III	6	9	0.421
Grade IV	3	2	0.625

^a^According to the guidelines for diagnosis and treatment of primary liver cancer issued by the National Health Commission of China in 2017.

**Table 3:** Primary and secondary outcomes.

Variable	Control group (n = 41)	TAP + LMA group (n = 42)	*P*-value
Postoperative time of readiness for discharge (days)	8 (5–13)	7 (5–11)	0.004
Postoperative apioid (sufentanil) requirement within 48 h (µg)	135.7 ± 20.1	102.8 ± 12.4	0.000
**NRS pain score**			
2 h after surgery	4.2 ± 1.4	3.3 ± 1.1	0.001
6 h after surgery	4.8 ± 1.4	3.8 ± 1.1	0.001
24 h after surgery	3.8 ± 1.1	3.1 ± 1.2	0.007
48 h after surgery	3.0 ± 1.1	2.3 ± 0.9	0.001
First mobilization off the bed (h)	43.8 ± 5.1	28.1 ± 5.5	0.000
Time to first flatus (h)	47.2 ± 7.6	32.7 ± 5.8	0.000
**QoR-40 score**			
1 day before surgery	194.5 ± 3.3	193.8 ± 3.1	0.323
1 day after surgery	169.5 ± 6.1	179.0 ± 5.6	0.000
3 days after surgery	176.0 ± 5.1	187.8 ± 5.1	0.000
Day of discharge	192.0 ± 5.0	193.6 ± 4.2	0.113
**Incidence of nausea/vomiting within 48 h**			
Nausea	6	4	0.475
Vomiting	3	1	0.294
Total cost for treatment (CNY)	84,434.0 ± 9,436.2	66,608.4 ± 6,268.4	0.000

The perioperative profiles of anesthesia and surgery in the two groups were summarized in Table 4. Blood glucose and lactate during surgery were both significantly lower in the TAP + LMA group than the control group. Patients in the TAP + LMA group required significantly less intraoperative anesthetic medicines (sevoflurane: 39.3 ± 6.5 ml vs. 54.6 ± 9.4 ml, remifentanil: 1,072.9 ± 190.4 µg vs. 1625.4 ± 264.8 µg, cis-atracurium: 21.5 ± 3.7 mg vs. 35.8 ± 4.5 mg) and less norepinephrine (864.5 ± 108.8 µg vs. 1,203.7 ± 191.1 µg) than those in the control group. During the implementation of controlled-low CVP, the requirement of dobutamine in the TAP + LMA group was significantly less than that of the control group (9.6 ± 2.4 mg vs. 11.2 ± 2.8 mg), while the requirement of nitroglycerin was similar between the two groups. Compared with the control group, the duration of hepatectomy and hepatic blood flow occlusion in the TAP + LMA group decreased by approximately 10 min and 5 min, respectively. No significant differences were observed between the two groups in duration of anesthesia or surgery. There were significant differences between the two groups regarding bleeding volume, infusion volume and urine volume. The time to removing ETT/LMA and the duration of PACU stay was statistically shorter in the TAP + LMA group than the control group. There were no statistical differences regarding perioperative complications between the two groups (Table 5). One patient in the control group experienced post-discharge wound infection and was hospitalized again 15 days later.

**Table 4:** Perioperative profiles of anesthesia and surgery.

Variable	Control group (n = 41)	TAP + LMA group (n = 42)	*P*-value
**Blood lactate (mmol/L)**			
t0	0.9 ± 0.2	0.9 ± 0.3	0.829
t1	0.9 ± 0.2	1.0 ± 0.2	0.180
t2	2.1 ± 0.5	1.6 ± 0.4	0.000
t3	1.5 ± 0.4	1.1 ± 0.2	0.000
**Blood glucose (mmol/L)**			
t0	5.1 ± 0.5	4.9 ± 0.6	0.068
t1	5.5 ± 0.5	5.2 ± 0.2	0.039
t2	8.4 ± 0.8	7.8 ± 0.7	0.001
t3	7.4 ± 1.0	7.1 ± 0.8	0.080
**Anesthetic requirements**			
Sevoflurane (ml)	54.6 ± 9.4	39.3 ± 6.5	0.000
Remifentanil (µg)	1625.4 ± 264.8	1,072.9 ± 190.4	0.000
Cis-atracurium (mg)	35.8 ± 4.5	21.5 ± 3.7	0.000
**Requirements of vasoactive drugs**			
Nitroglycerin (mg)	2.8 ± 0.6	2.7 ± 0.7	0.467
Dobutamine (mg)	11.2 ± 2.8	9.6 ± 2.4	0.006
Norepinephrine (µg)	1,203.7 ± 191.1	864.5 ± 108.8	0.000
Duration of anethesia (min)	268.3 ± 33.5	255.9 ± 35.3	0.103
Duration of operation (min)	222.8 ± 32.2	209.3 ± 35.2	0.073
Duration of hepatectomy (min)	122.9 ± 18.2	114.4 ± 15.4	0.023
Duration of hepatic blood flow occlusion (min)	26.5 ± 5.2	21.5 ± 3.5	0.000
Bleeding volume (ml)	480.5 ± 90.7	395.2 ± 68.8	0.000
Infusion volume (ml)	1839.0 ± 374.8	1631.0 ± 215.8	0.003
Urine volume (ml)	414.6 ± 86.8	500.0 ± 96.9	0.000
Time to removing ETT or LMA (min)	32.8 ± 4.5	19.3 ± 3.9	0.000
PACU stay (min)	114.0 ± 14.2	95.6 ± 11.4	0.000

t0 = before anesthesia, t1 = at surgical exploration, t2 = at the conclusion of surgery, t3 = at departure from PACU.

**Table 5:** Complications related to anesthesia and surgery.

Complications	Control group (n = 41)	TAP + LMA group (n = 42)	*P*-value
**LMA or ETI-related**			
Intraoperative reflux/aspiration	0	0	1.000
Intraoperative airway obstruction	0	0	1.000
Postoperative sore throat	6	2	0.128
Postoperative hoarse voice	1	0	0.309
**TAP block or local infiltration-related**			
Systemic poisoning from local anesthetic	0	0	1.000
Abdominal hematoma	0	0	1.000
Abdominal nerve injury	0	0	1.000
Abdominal visceral injury	0	0	1.000
**Surgery-related**			
Intraperitoneal hemorrhage	0	0	1.000
Ileus	0	0	1.000
Lung infection	0	0	1.000
Poor wound healing	1	0	0.309
Bile leakage	0	0	1.000
Readmission within 30 days	1	0	0.309

## Discussion

The incidence of primary liver cancer ranks fourth among all kinds of malignant tumors, while the mortality rate ranks third^14^. China is the country of the highest incidence of liver cancer, where the annual number of new cases and deaths account for more than half of the total cases of the world^14,15^. Resection of tumor is the most crucial component among the comprehensive treatments for liver cancer^16^.However, hepatectomy, especially open hepatectomy, usually induces severe stress and inflammation, which may delay postoperative recovery and increase complications as well as medical cost. ERAS guidelines promulgated by the International ERAS Society in 2016 recommended that the peripheral nerve block, but not epidural block, should be considered as anesthesia and analgesia for open hepatectomy to reduce stress and improve recovery^17^. As an alternative to ETI, LMA can be used safely during abdominal surgery and general anesthesia. It can reduce stress response, accelerate recovery and make the patient more comfortable^18,19^. In this trial, we confirmed that the anesthesia protocol of the TAP block combined with LMA can be safely used in open hepatectomy with an ERAS program and has satisfactory outcomes in alleviating stress and pain, promoting recovery, and reducing medical cost.

In this study, we chose to place TAP block before surgery, in order to control surgical stress more effectively, and meanwhile reduce the dose of anesthetics, opioids and muscle relaxants, which would help patients recover faster after surgery. Arterial pressure, heart rate and blood glucose are the most common indicators representing the intensity of stress response. In this study, we adjusted arterial pressure, heart rate and anesthetic depth in both groups to the same level during surgery, and recorded the dosage of narcotics (which reflected the stress level indirectly). As a result, the protocol of TAP block combined with LMA greatly reduced the dosages of narcotics (consisting of hypnotics, opioids and muscle relaxants). Meanwhile, the concentration of blood glucose after surgery was significantly lower in the TAP + LMA group than in that of control group. Blood lactate can reflect stress intensity and perfusion of organ and tissue. Some recent studies have reported that the level of blood lactate is significantly correlated with hospitalization duration, incidence of complications and risk of death^20,21^. Our trial showed lower lactate and more urine in the patients of TAP + LMA group than in the control group. Besides, we found that the TAP + LMA scheme was more conducive to the implementation of controlled-low CVP. The duration of hepatectomy, duration of hepatic blood flow occlusion, blood loss, infusion volume and dosages of vasoactive drugs were less in the TAP + LMA group than those of the control group. These outcomes probably resulted from the mutually beneficial effects of the TAP + LMA protocol, that is, optimal anesthetic efficacy as well as minimal interference of the internal environment.

In this study, we proved that the anesthesia protocol of TAP + LMA could greatly enhance recovery after open liver surgery and improve the comfort of patients during hospitalization. Compared to the control group, patients of the TAP + LMA group had shorter time to anesthesia recovery, first flatus, first mobilization, hospital discharge and experienced less pain. This indicated the efficacy of the TAP + LMA protocol used in the implementation of ERAS for liver cancer surgery, which was consistent with the outcomes from previous studies related to the TAP block^22,23^. Since reported firstly in 2000, the 40-item quality of recovery (QoR-40) survey has proved to be a reliable and sensitive measure for assessing the quality of postoperative recovery worldwide^12,13,24^. It pays more attention to the patients’ subjective feelings and is done through a questionnaire of 40 items, characterized by Patient-reported Quality (PRO), which is different from Doctor-reported Quality (DRO). In this study, there was a significant difference in the QoR-40 scores between the two groups at 1 and 3 days after surgery. Using the TAP + LMA protocol made the patients more comfortable during recovery. In the implementation of ERAS, the TAP + LMA protocol potentially reduced the hospitalization cost for patients undergoing open hepatectomy by almost 18,000 CNY, and didn’t increase complications related to anesthesia and surgery. This convinced us that the TAP + LMA protocol could be considered as an ideal anesthesia method for open liver surgery.

The original purpose of this randomized controlled trial was to explore the optimal anesthesia protocol for patients undergoing open hepatectomy with an ERAS program, from the perspective of anesthesiology. Ultrasound imaging allows more accurate administration of TAP block, which ensures a satisfactory block outcome and minimizes the risk of visceral organs injury^25^. The TAP block has little impact on visceral function and almost no contraindications, so it can be used routinely as analgesia for abdominal surgery^9^. The TAP block can also be individually implemented depending on the type of surgery, the location and size of incision and the condition of patient. Referring to previous studies^26,27^, in this study we mixed ropivacaine with dexamethasone in the TAP block in order to extend the analgesic duration of the local anesthetic. Although the ropivacaine has a short half life of 8–10 h in blood, it may have a longer analgesic effect if it is used in a direct nerve block, especially by addition of dexamethasone. And the result showed a significant difference in the opioid consumption within 48 h postoperatively. The insertion of LMA is very easy and does not require a special position, intubation tools or muscle relaxants^18^. Therefore, the combined use of the TAP block and double-tube LMA in patients undergoing open hepatectomy with an ERAS program is simple to perform and has satisfying clinical outcomes.

Recently, two randomized trials reported that minimally invasive radical surgery of cervical or bladder cancer did not decrease the rates of disease-free survival and overall survival compared with open surgery^28,29^. Compared with open surgery, laparoscopic surgery gains faster early recovery for smaller size of somatic wound. However, laparoscopic surgery requires artificial pneumoperitoneum, special positioning and longer operation time, which may have adverse effects on operative recovery and long-term prognosis. TAP block can provide perfect wound analgesia for both laparoscopic and open surgery and can be easily performed in 10 min by an experienced anesthesiologist. Thus, surgeons no longer need to hesitate when choosing laparoscopic or laparotomy surgery for patients in the context of wide application of an ultrasound-guided TAP block. In short, the protocol of TAP block combined with LMA is a promising anesthesia method for patients undergoing abdominal surgery with an ERAS program.

## Limitations

This study has certain limitations. We chose open liver surgery only to assess the outcomes of the TAP + LMA protocol and did not know whether laparoscopic surgery had the same effects. The TAP block scheme in this study was designed for a reverse “L” incision in the right upper abdomen, the most commonly used type of incision for open liver surgery. Actually, other types of incisions for open liver surgery may be used such as Mercedes incision, median longitudinal incision and combined thoraco-abdominal incision. Satisfactory analgesia for these incisions could be achieved by changing the injection site of the TAP block or adding other peripheral nerve blockades. We limited the subjects to patients of clinical stages I and II, so the outcome and safety of the TAP + LMA protocol for surgical treatment of complex liver cancer and/or extremely large tumors is not yet known. In addition, we did not test plasma concentration of ropivacaine and gained little insight about the pharmacokinetics of ropivacaine during a TAP block.

## Conclusion

In summary, combined usage of a TAP block and LMA is a simple and safe anesthesia protocol during implementing ERAS programs for patients with primary liver cancer. It can alleviate surgical stress, accelerate recovery and reduce medical cost.

## Supplementary information

Supplementary file 1

Supplementary file 2

Supplementary file 3

## Abbreviations

TAP: Transversus abdominis plane
LMA: Laryngeal mask airway
ERAS: Enhanced recovery after surgery
ETI: Endotracheal intubation
ETT: Endotracheal tube
CT: Computerized tomography
MRI: Magnetic resonance imaging
ICU: Intensive care unit
NI: Narcotrend Index
CVP: Central venous pressure
PCIA: Patient-controlled intravenous analgesia
PACU: Post-anesthesia care unit
NRS: Numerical rating scale
QoR: Quality of recovery
PRO: Patient-reported quality
DRO: Doctor-reported quality
