# Supplementary material for: Combined use of transversus abdominis plane block and laryngeal mask airway during implementing ERAS programs for patients with primary liver cancer: a randomized controlled trial
Source: Sci Rep. 2020 Sep 10;10:14892. doi: 10.1038/s41598-020-71477-x (PMC7483533; doi:10.1038/s41598-020-71477-x)
Supplement: Supplementary file 2 [file 41598_2020_71477_MOESM2_ESM.docx]

**Combined use of transversus abdominis plane block and laryngeal mask airway during implementing ERAS programs for patients with primary liver cancer：study protocol for a randomized controlled trial**

Hai-ming Huang^a#^, Rui-xia Chen^a#^, Lin-mei Zhu^b^, Wen-shuai Zhao^a^, Xi-jiu Ye^a^, Jian-wei Luo^a^, Fu-ding Lu^a^, Lei Zhang^c^, Xue-ying Yang^a^, Yuan Yuan^a^, Jun Cao^c*^

^a^ Department of Anesthesiology, Sun Yat-sen Memorial Hospital, Sun Yat-sen University, Guangzhou, Guangdong, 510120, China

^b^ Department of Obstetrics and Gynecology, Clifford Hospital, Guangzhou University of Chinese Medicine, Guangzhou, Guangdong, 511495, China

^c^ Department of Hepatobiliopancreatic Surgery, Sun Yat-sen Memorial Hospital, Sun Yat-sen University, Guangzhou, Guangdong, 510120, China

^#^These authors contributed equally to this work.

^*^ Corresponding Author: Prof. Jun Cao, Department of Hepatobiliopancreatic Surgery, Sun Yat-sen Memorial Hospital, Sun Yat-sen University, NO. 107, Yanjiangxi Road, Guangzhou, Guangdong, 510120, China; E-mail: 307423373@qq.com

**Study design**

This prospective, evaluator-blinded, randomized, controlled parallel-arm trial will be conducted at Sun Yat-sen Memorial Hospital, Sun Yat-sen University, China. The study protocol is approved by the Medical Ethics Committee of Sun Yat-sen Memorial Hospital of Sun Yat-sen University (Approval number: 201806). All procedures will be performed in accordance with the declaration of Helsinki. Written informed consent was obtained from every patient before randomization.

**Patients**

Patients with primary liver cancer who is scheduled for open hepatectomy with ERAS programs (**Table 1**) will be evaluated for eligibility. Inclusion criteria are as follows: age between 18 and 65 years; body mass index of 18.5-28 kg/m^2^; number of tumor less than 3; tumor’s maximum diameter or sum of diameters smaller than 10 cm; class A or B of Child-Pugh liver function; tumor’s clinical stage as I or II; the remaining liver volume/standard liver volume ratio of >40%; no difficulty of intubation or inserting an LMA; American Society of Anesthesiologist physical status classification between I and III. Exclusion criteria are as follows: presence of comorbidities such as diabetes, pathological cardiopulmonary disease and renal insufficiency; a New York Heart Association class of heart function of III or greater; intrahepatic vascular invasion or extrahepatic metastasis of tumor seen by computerized tomography (CT) or magnetic resonance imaging (MRI); having received chemotherapy or radiotherapy before surgery; having undergone abdominal surgery; infection of the site of TAP block; allergy to any study medication; a history of alcohol or drug abuse; difficulty of communication with family or hospital staff. After randomization, patients who experience a failure of TAP block or LMA ventilation, suffer systemic poisoning from local anesthetic, are found to have extensive metastasis of tumor after laparotomy, lose blood over 1000 ml during surgery, undergo a long operating time of over 6 hours (hr), need to be transferred into intensive care unit (ICU) after surgery, or refuse to go on participating in this trial will br excluded from the analysis.

**Table 1 The ERAS program adopted in this trial**

| **Preoperative procedures** |
| --- |
| **·**Preoperative assessment, education and psychological counseling by talk, text and caption |
| **·**Preparation before admission: cessation of smoking and alcohol for 14 days, quick walk for 30 minutes daily, oral enteral nutritional powder if plasma albumin ≤30g/L, infuse red blood cell if Hb <70g/L |
| **·**No preoperative use of sedatives or anticholinergic drugs |
| **·**No bowel preparation, fasting for 6hr, and oral 10% glucose 500ml 2 hr before surgery |
| **·**No routine placement of gastric tube; if necessary, remove it as soon as possible |
| **·**Insert the urinary tube under lidocaine gel blockade and remove it within 48 hr |
| **·**Antibiotic prophylaxis 30min before surgery |
| **·**Use heparin to prevent deep vein thrombosis for high risk patients and monitor coagulation |
| **Intraoperative procedures** |
| **·**Continuously monitor body temperature and maintain its stability by warmed blanket, warmed infusion and preheated peritoneal irrigation |
| **·**Infuse crystal liquid mainly and restrict the volume (CVP ≤10 mmHg); rapidly infuse 200~300ml colloidal fluid if severe hypotension occurs during the implementation of controlled-low CVP |
| **·**No routine placement of peritoneal drainage tube; if necessary, remove it within 48 hours |
| **·**Anesthesia method: ETI general anesthesia for the control group, LMA general anesthesia combined with a subcostal TAP block for the TAP+LMA group |
| **·**Use of short-acting anesthetics (i.e. remifentanil, sufentanil, propofol and sevoflurane) |
| **·**Monitor anesthetic depth and maintain its stability |
| **·**Intravenous tropisetron 10mg for preventing postoperative nausea and vomiting |
| **Postoperative procedures** |
| **·**Multimodal postoperative analgesia: a. regional block (local wound infiltration for the control group and a subcostal TAP block for the TAP+LMA group); b. patient-controlled intravenous analgesia (PCIA) with sufentanil; c. intravenous parecoxib 40mg twice daily for 3 days; d. oral analgesic |
| **·**Assess pain intensity with numerical rating scale (NRS) and inject additional analgesic when NRS score≥5 |
| **·**Intravenouslydrip dexamethasone 5mg daily for 3 days to alleviate inflammation |
| **·**Treatment of postoperative nausea and vomiting |
| **·**Remove drainage tube and urinary tube as soon as possible |
| **·**Early oral intake: a little water on the day of surgery; liquid diet on 1^st^ postoperative day; semi-liquid  diet on 2^nd^ postoperative day; normal diet from 3^rd^ postoperative day on |
| **·**Early mobilization: exercise in bed on the day of surgery; walk in the ward at least twice on 1^st^ postoperative day; walk at least 4 times on 2^nd^ postoperative day; continuously walk from 3^rd^ postoperative day on |
| **·**Use low molecular weight heparin to prevent deep vein thrombosis |

**Randomization and blinding**

This study is an observer-blinded, randomized control trial. Randomization, stratified by surgeons, will be performed by independent personnel (Zhu L), through two random digit tables generated by computer software. The random digit and the information of group allocation for every enrolled patient is sealed with an opaque envelope. Two anesthesiologists (Chen R, Yang X) will perform all the intraoperative assessments, and two other anesthesiologists (Yuan Y, Lu F) will perform all the postoperative assessments; two surgeons (Cao J, Zhang L) will assess if the patients achieve the standard of hospital discharge, and two independent persons (Zhao W, Ye X) will perform the statistical analysis of the data. All these investigators are blinded to the group allocation. All procedures of TAP blockades and intubation or insertion of LMAs will be performed by the same anesthesiologist (Huang H), who knows the group allocation. The flow diagram for this trial is summarized in **Figure 1**.

Identify patients with primary liver cancer

scheduled for open hepatectomy with ERAS programs

Review pre-operative assessment to determine eligibility

Obtaine informed consent

Perform randomization

(n=48)

Experimental group (TAP+LMA group):

a subcostal TAP block combined with LMA anesthesia

Control group:

general anesthesia with endotracheal intubation

Study end-points:

postoperative time of readiness for discharge; postoperative pain intensity;

time to first flatus; quality of recovery; complications; overall medical cost

**Figure 1 Flow diagram for this trial**

**Interventions**

Patients in both groups will receive the same ERAS program except for the different interventions. For the control group, patients will be treated with endotracheal intubation for ventilation after anesthesia induction, a preoperative TAP block with 40 ml normal saline as control and local wound infiltration with 40 ml solution of ropivacaine 3 mg/kg plus dexamethasone 0.1 mg/kg at the conclusion of surgery. On the contrary, patients in the TAP+LMA group will have an LMA inserted for ventilation after induction, have a TAP block with 40ml solution of ropivacaine 3 mg/kg plus dexamethasone 0.1 mg/kg, and receive local wound infiltration with 40 ml normal saline at the conclusion of surgery. Guided by ultrasound, a subcostal TAP block will be performed bilaterally at the parasternal line and at the anterior axillary line, with 10ml solution injected in each site (**Figure 2**).


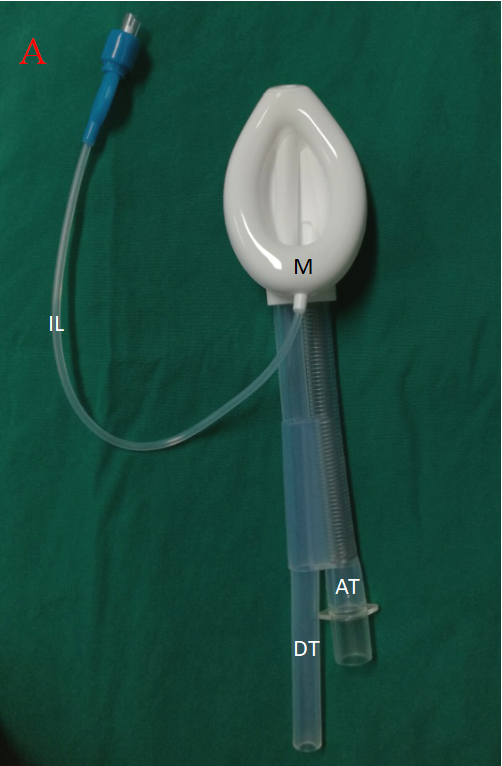

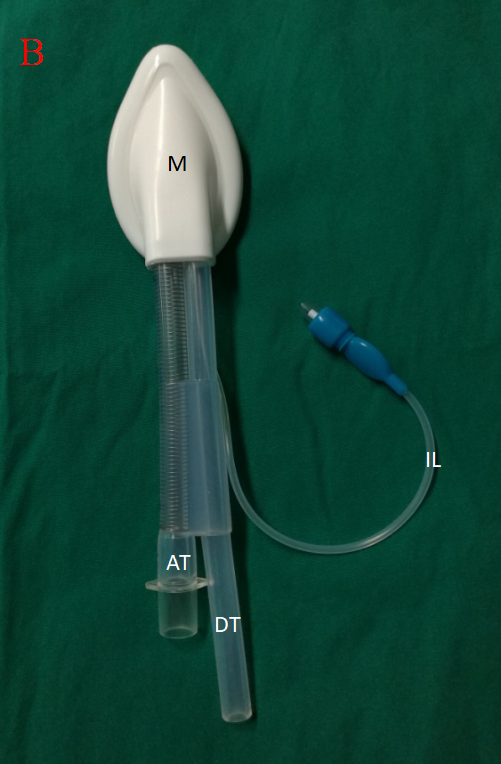


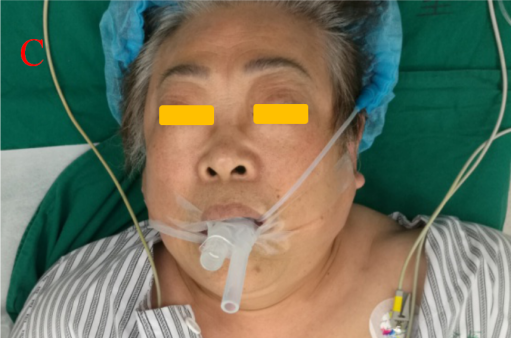

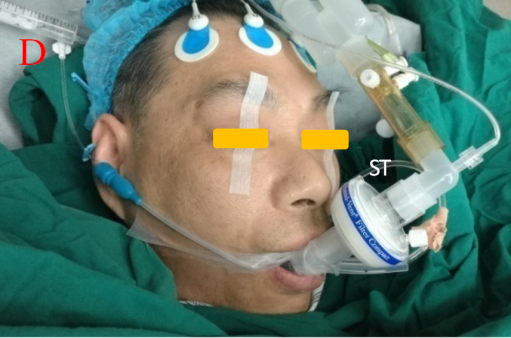


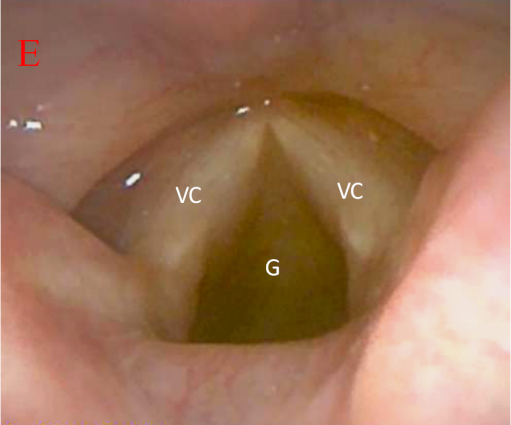

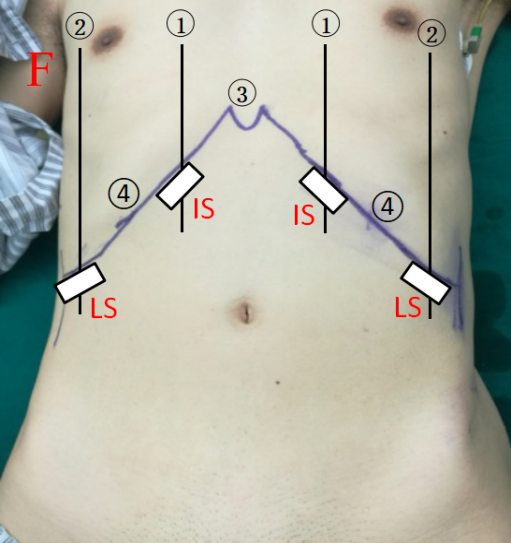


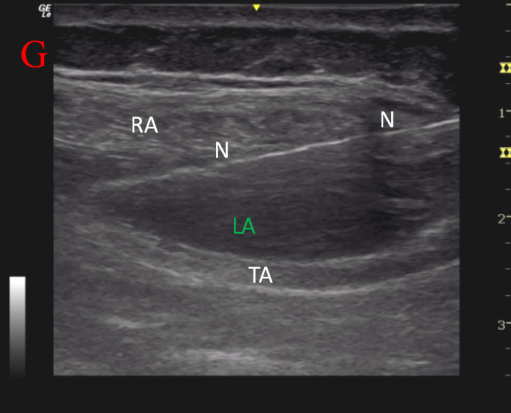

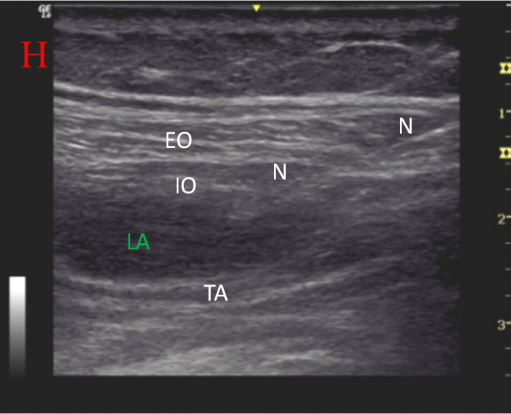


**Figure 2 Procedures of LMA ventilation and subcostal TAP block**

1. Double-tube LMA: ventral side; B. Double-tube LMA: back side; C. Placement of LMA; D.LMA ventilation and esophageal drainage; E.LMA positioning by fiberoptic bronchoscopy; F. positions of ultrasound probe for TAP block; G. Ultrasound image of TAP block: inner site; H. Ultrasound image of TAP block: lateral site.

(AT: airway tube, DT: esophageal drainage tube, M: mask, IL: inflation line, ST: stomach tube, G: glottal, VC: vocal cord, ①: parasternal line, ②: anterior axillary line, ③: xiphoid, ④: costal arch, white rectangle: ultrasound probe, IS: inner site, LS: lateral site, N: needle, LA: local anesthetic, RA: rectus abdominis, TA: transversus abdominis, EO: obliquus externus abdominis, IO: obliquus internus abdominis)

**Anesthesia and analgesia**

While in the operating room, all enrolled patients will be continuously monitored for standard measures including electrocardiogram, heart rate, non-invasive arterial blood pressure and oxygen saturation. Monitoring of Narcotrend Index (NI) is used for assessing patients’ depth of consciousness. Placement of a peripheral vein catheter is established for infusion. Radial artery catheterization is performed to monitor invasive arterial pressure and to measure blood gas.

After induction of total intravenous anesthesia with propofol/sufentanil/cis-atracurium, positive pressure ventilation will be performed in the patients of control group by ETI and in the patients of TAP+LMA group by LMA. Auscultation of respiratory tone, observation of respiratory parameters and examination with a flexible fiberoptic bronchoscopy is used to determine whether the laryngeal mask is in correct position. If necessary, gastric tubes will be placed with the help of a laryngoscope in the patients of control group or through the esophageal drainage tube of the LMA in the patients of the TAP+LMA group. Then, the procedures of ultrasound-guided subcostal TAP block will be implemented as described above.

Maintenance of anesthesia is achieved by administration of sevoflurane, remifentanil and cis-atracurium. Dosages of anesthetic drugs is adjusted to maintain arterial blood pressure and heart rate fluctuating in the range of -20% to +20% of the base value and NI fluctuating in the range of 37 to 56. If necessary, cardiovascular active drugs (i.e. norepinephrine, atropine, esmolol, nicardipine) will be used to maintain stable hemodynamics. Controlled-low Central Venous Pressure (CVP) technology is adopted to maintain CVP between 0 and 5 cmH_2_O during hepatectomy. This is done by restriction of fluid, control of anesthesia depth and stress response, adjustment of patients’ position, and application of vasoactive drugs (nitroglycerin and dobutamine). The CVP value will be restored to normal after completing the hepatectomy. Allogeneic blood transfusion will be administered if hemoglobin was less than 70 g/L. Body temperature is continuously monitored and maintained within 36～37℃ by multiple approaches including warmed infusion, application of electric blanket and preheated peritoneal irrigation.

At the conclusion of surgery, tropisetron 10mg is administered to prevent postoperative nausea and vomiting and sufentanil 0.1ug/kg is administered to alleviate remifentanil-induced hyperalgesia. Local wound infiltration is performed with different solution in the two groups, as described above. Multimodal analgesia is used postoperatively in both groups, including regional block, patient-controlled intravenous analgesia (PCIA) with sufentanil, intravenous parecoxib 40 mg twice per day for 3 days and oral analgesic.

**Study end-points**

The primary end-point of the study is postoperative time of readiness for hospital discharge. The discharge criteria include: pain score less than 4, solid diet without infusion, normal bowel movement, well-healed wound, normal organ function, normal count of white blood cell (WBC), no fever, life independence.

The secondary end-points are postoperative analgesic requirement within 48 hr, time to first flatus and overall medical cost. Blood glucose and lactate will be tested before anesthesia (t0), at surgical exploration (t1), at the conclusion of surgery (t2) and at departure from the post-anesthesia care unit (PACU) (t3). Dosages of anesthetics and vasoactive drugs, surgery duration, anesthesia duration, hepatectomy duration, duration of hepatic blood flow occlusion, bleeding volume, infusion volume and urine volume are recorded.

The postoperative pain intensity of the surgical site is assessed with the numerical rating scale (NRS), in which 0 represented no pain and 10 represented the most severe pain. Postoperative variables such as time to removing ETT or LMA, duration of PACU stay, time to first mobilization off the bed, incidence of nausea/vomiting within 48 hr and complications are also recorded and compared. The 40-item quality of recovery (QoR-40) survey is performed before surgery, at 1 and 3 days after surgery, and on the day of discharge, referred to other studies^[12, 13]^ and described as **the following figure**.

| **QoR-40 Questionnaire** | | | | | | | | |
| --- | --- | --- | --- | --- | --- | --- | --- | --- |
| Date:____/____/____ | Preoperative:_______ | | | Postoperative:______ | | | Study #:______ | |
| **PART A** | | | | | | | | |
| ***How have you been feeling in the last 24 hours?*** | | | | | | | | |
| (1 to 5, where: 1 = none of the time [poor], 2 = sometimes, 3 = often, 4 = most of the time, 5 = all of the time [excellent]) | | | | | | | | |
|  | | none of  the time | sometimes | | often | most of  the time | | all of the time |
| 1.Able to breathe easy | | 1 | 2 | | 3 | 4 | | 5 |
| 2.Being able to enjoy food | | 1 | 2 | | 3 | 4 | | 5 |
| 3.Have a good sleep | | 1 | 2 | | 3 | 4 | | 5 |
| 4.Feeling rested | | 1 | 2 | | 3 | 4 | | 5 |
| 5.Feeling comfortable | | 1 | 2 | | 3 | 4 | | 5 |
| 6.Having a general feeling  of well-being | | 1 | 2 | | 3 | 4 | | 5 |
| 7.Feeling in control | | 1 | 2 | | 3 | 4 | | 5 |
| 8.Able to write | | 1 | 2 | | 3 | 4 | | 5 |
| 9.Have normal speech | | 1 | 2 | | 3 | 4 | | 5 |
| 10.Able to wash, brush teeth orshave | | 1 | 2 | | 3 | 4 | | 5 |
| 11.Able to look after own appearance | | 1 | 2 | | 3 | 4 | | 5 |
| 12.Able to return to work or usual home activities | | 1 | 2 | | 3 | 4 | | 5 |
| 13.Able to communicate with hospital staff (when in hospital) | | 1 | 2 | | 3 | 4 | | 5 |
| 14.Able to communicate with family or friends | | 1 | 2 | | 3 | 4 | | 5 |
| 15.Getting support from doctors (when in hospital) | | 1 | 2 | | 3 | 4 | | 5 |
| 16. Getting support from nurses (when in hospital) | | 1 | 2 | | 3 | 4 | | 5 |
| 17. Having support from family or friends | | 1 | 2 | | 3 | 4 | | 5 |
| 18.Able to understand instructions or advice | | 1 | 2 | | 3 | 4 | | 5 |
| **PART B** | | | | | | | | |
| ***Have you had any of the following in the last 24 hours?*** | | | | | | | | |
| (5 to 1, where: 5 = none of the time [poor], 4 = sometimes, 3 = often, 2 = most of the time, 1 = all of the time [excellent]) | | | | | | | | |
|  | | none of  the time | sometimes | | often | most of  the time | | all of the time |
| 19.Moderate pain | | 5 | 4 | | 3 | 2 | | 1 |
| 20.Severe pain | | 5 | 4 | | 3 | 2 | | 1 |
| 21.Headache | | 5 | 4 | | 3 | 2 | | 1 |
| 22.Muscle pain | | 5 | 4 | | 3 | 2 | | 1 |
| 23.Backache | | 5 | 4 | | 3 | 2 | | 1 |
| 24.Sore throat | | 5 | 4 | | 3 | 2 | | 1 |
| 25.Sore mouth | | 5 | 4 | | 3 | 2 | | 1 |
| 26.Nausea | | 5 | 4 | | 3 | 2 | | 1 |
| 27.Vomitting | | 5 | 4 | | 3 | 2 | | 1 |
| 28.Dry retching | | 5 | 4 | | 3 | 2 | | 1 |
| 29.Feeling restless | | 5 | 4 | | 3 | 2 | | 1 |
| 30.Shaking or twitching | | 5 | 4 | | 3 | 2 | | 1 |
| 31.Shivering | | 5 | 4 | | 3 | 2 | | 1 |
| 32.Feeling too cold | | 5 | 4 | | 3 | 2 | | 1 |
| 33.Feeling dizzy | | 5 | 4 | | 3 | 2 | | 1 |
| 34. Feeling confused | | 5 | 4 | | 3 | 2 | | 1 |
| 35.Difficulty falling asleep | | 5 | 4 | | 3 | 2 | | 1 |
| 36.Bad dreams | | 5 | 4 | | 3 | 2 | | 1 |
| 37.Feeling angry | | 5 | 4 | | 3 | 2 | | 1 |
| 38.Feeling anxious | | 5 | 4 | | 3 | 2 | | 1 |
| 39.Feeling depressed | | 5 | 4 | | 3 | 2 | | 1 |
| 40.Feeling alone | | 5 | 4 | | 3 | 2 | | 1 |

**Statistical analysis and sample size**

Statistical analyses will be performed with SPSS software version 19.0 (IBM Corporation, Armonk, NY, USA). Continuous variables are expressed as mean ± standard deviation and tested if they are normally distributed. Variables with normal distribution are compared between groups using a 2-sample Student’s *t*-test. Data without normal distribution are analyzed using non-parametric test. Blood glucose, lactate, the NRS pain score and the QoR-40 score will be compared between groups using repeated-measures analysis of variance (ANOVA). Categorical data is expressed as ratio or frequency and analyzed using Chi-square test or Fisher’s exact test. Two-tailed *P*<0.05 indicates statistical significance.

Based on our preliminary experiment consisting of 24 patients, the sample size for this trial is calculated using SAS software version 9.4 (SAS Institute Inc., Iowa City, IA, USA). The postoperative time of readiness for hospital discharge in the control group was (9 ± 2.5) days, while that of the TAP+LMA group was (7.5 ± 2.0) days. Accordingly, 42 patients are required per group to achieve 85% statistical power (1-β) at a significance level (α) of 0.05. To account for a 15% drop-out rate, we will enrolled 48 patients per group finally.
