# Supplementary material for: Combined use of transversus abdominis plane block and laryngeal mask airway during implementing ERAS programs for patients with primary liver cancer: a randomized controlled trial
Source: Sci Rep. 2020 Sep 10;10:14892. doi: 10.1038/s41598-020-71477-x (PMC7483533; doi:10.1038/s41598-020-71477-x)
Supplement: Supplementary file 3 [file 41598_2020_71477_MOESM3_ESM.docx]

**Combined Use of Transversus Abdominis Plane Block and Laryngeal Mask Airway during Implementing ERAS Programs for Patients with Primary Liver Cancer: a Randomized Controlled Trial**

Hai-ming Huang^a#^, Rui-xia Chen^a#^, Lin-mei Zhu^b^, Wen-shuai Zhao^a^, Xi-jiu Ye^a^, Jian-wei Luo^a^, Fu-ding Lu^a^, Lei Zhang^c^, Xue-ying Yang^a^, Yuan Yuan^a^, Jun Cao^c*^

^a^ Department of Anesthesiology, Sun Yat-sen Memorial Hospital, Sun Yat-sen University, Guangzhou, Guangdong, 510120, China

^b^ Department of Obstetrics and Gynecology, Clifford Hospital, Guangzhou University of Chinese Medicine, Guangzhou, Guangdong, 511495, China

^c^ Department of Hepatobiliopancreatic Surgery, Sun Yat-sen Memorial Hospital, Sun Yat-sen University, Guangzhou, Guangdong, 510120, China

^#^These authors contributed equally to this work.

^*^ Corresponding Author: Prof. Jun Cao, Department of Hepatobiliopancreatic Surgery, Sun Yat-sen Memorial Hospital, Sun Yat-sen University, NO. 107, Yanjiangxi Road, Guangzhou, Guangdong, 510120, China; E-mail: 307423373@qq.com

**Supplementary material:**

| **QoR-40 Questionnaire** | | | | | | | | |
| --- | --- | --- | --- | --- | --- | --- | --- | --- |
| Date:____/____/____ | Preoperative:_______ | | | Postoperative:______ | | | Study #:______ | |
| **PART A** | | | | | | | | |
| ***How have you been feeling in the last 24 hours?*** | | | | | | | | |
| (1 to 5, where: 1 = none of the time [poor], 2 = sometimes, 3 = often, 4 = most of the time, 5 = all of the time [excellent]) | | | | | | | | |
|  | | none of  the time | sometimes | | often | most of  the time | | all of the time |
| 1.Able to breathe easy | | 1 | 2 | | 3 | 4 | | 5 |
| 2.Being able to enjoy food | | 1 | 2 | | 3 | 4 | | 5 |
| 3.Have a good sleep | | 1 | 2 | | 3 | 4 | | 5 |
| 4.Feeling rested | | 1 | 2 | | 3 | 4 | | 5 |
| 5.Feeling comfortable | | 1 | 2 | | 3 | 4 | | 5 |
| 6.Having a general feeling  of well-being | | 1 | 2 | | 3 | 4 | | 5 |
| 7.Feeling in control | | 1 | 2 | | 3 | 4 | | 5 |
| 8.Able to write | | 1 | 2 | | 3 | 4 | | 5 |
| 9.Have normal speech | | 1 | 2 | | 3 | 4 | | 5 |
| 10.Able to wash, brush teeth orshave | | 1 | 2 | | 3 | 4 | | 5 |
| 11.Able to look after own appearance | | 1 | 2 | | 3 | 4 | | 5 |
| 12.Able to return to work or usual home activities | | 1 | 2 | | 3 | 4 | | 5 |
| 13.Able to communicate with hospital staff (when in hospital) | | 1 | 2 | | 3 | 4 | | 5 |
| 14.Able to communicate with family or friends | | 1 | 2 | | 3 | 4 | | 5 |
| 15.Getting support from doctors (when in hospital) | | 1 | 2 | | 3 | 4 | | 5 |
| 16. Getting support from nurses (when in hospital) | | 1 | 2 | | 3 | 4 | | 5 |
| 17. Having support from family or friends | | 1 | 2 | | 3 | 4 | | 5 |
| 18.Able to understand instructions or advice | | 1 | 2 | | 3 | 4 | | 5 |
| **PART B** | | | | | | | | |
| ***Have you had any of the following in the last 24 hours?*** | | | | | | | | |
| (5 to 1, where: 5 = none of the time [poor], 4 = sometimes, 3 = often, 2 = most of the time, 1 = all of the time [excellent]) | | | | | | | | |
|  | | none of  the time | sometimes | | often | most of  the time | | all of the time |
| 19.Moderate pain | | 5 | 4 | | 3 | 2 | | 1 |
| 20.Severe pain | | 5 | 4 | | 3 | 2 | | 1 |
| 21.Headache | | 5 | 4 | | 3 | 2 | | 1 |
| 22.Muscle pain | | 5 | 4 | | 3 | 2 | | 1 |
| 23.Backache | | 5 | 4 | | 3 | 2 | | 1 |
| 24.Sore throat | | 5 | 4 | | 3 | 2 | | 1 |
| 25.Sore mouth | | 5 | 4 | | 3 | 2 | | 1 |
| 26.Nausea | | 5 | 4 | | 3 | 2 | | 1 |
| 27.Vomitting | | 5 | 4 | | 3 | 2 | | 1 |
| 28.Dry retching | | 5 | 4 | | 3 | 2 | | 1 |
| 29.Feeling restless | | 5 | 4 | | 3 | 2 | | 1 |
| 30.Shaking or twitching | | 5 | 4 | | 3 | 2 | | 1 |
| 31.Shivering | | 5 | 4 | | 3 | 2 | | 1 |
| 32.Feeling too cold | | 5 | 4 | | 3 | 2 | | 1 |
| 33.Feeling dizzy | | 5 | 4 | | 3 | 2 | | 1 |
| 34. Feeling confused | | 5 | 4 | | 3 | 2 | | 1 |
| 35.Difficulty falling asleep | | 5 | 4 | | 3 | 2 | | 1 |
| 36.Bad dreams | | 5 | 4 | | 3 | 2 | | 1 |
| 37.Feeling angry | | 5 | 4 | | 3 | 2 | | 1 |
| 38.Feeling anxious | | 5 | 4 | | 3 | 2 | | 1 |
| 39.Feeling depressed | | 5 | 4 | | 3 | 2 | | 1 |
| 40.Feeling alone | | 5 | 4 | | 3 | 2 | | 1 |

**Figure S1 The 40-item quality of recovery (QoR-40) Questionnaire**
